# Multiple Sclerosis: From Molecules to Treatment

**DOI:** 10.3390/ijms14047598

**Published:** 2013-04-08

**Authors:** Simon A Broadley

**Affiliations:** 1 School of Medicine, Gold Coast Campus, Griffith University, QLD 4222, Queensland, Australia; E-Mail: simon.broadley@griffith.edu.au; Tel.: +61-7-5678-0702; Fax: +61-7-5678-0708; 2 Department of Neurology, Gold Coast Hospital, Southport QLD 4215, Queensland, Australia

**Keywords:** multiple sclerosis, genetics, epidemiology, treatment, pathophysiology, immunology

## Abstract

The treatment of multiple sclerosis has been radically transformed over the past 20 years and this special issue of IJMS, focusing on the molecular aspects of the disease, highlights the growing conformity of the various investigative approaches. It is a very exciting time to be involved in the research of this disease.

The worlds of science and medicine have been aware of multiple sclerosis (MS) as a distinct clinical [1] and pathological entity [2] for 175 years. The key pathological features were described early on and remained essentially unchanged except for a natural increase in precision and detail of the changes described as technology has improved [3]. Recent reassessment of the pathological features has begun to highlight apparent heterogeneity even within individual cases [4]. This of course comes as no surprise to clinicians: it was Charcot who first commented on the multitude of clinical features and highly variable clinical course that MS can present [1]. This has led to difficulties with diagnosis in some cases and has necessitated a very careful approach to the design of clinical trials [5]. Formal diagnostic criteria have been progressively refined and there is now a growing reliance upon MRI features, but the core clinical features of dissemination of clinical lesions in time and space remain central to the diagnosis [6].

Careful epidemiological work over the past 60 years has identified that both genetic and environmental factors influence the risk of developing MS. A familial association in MS was demonstrated conclusively early on [7] and in the last five years, in excess of 60 genetic loci with definitive evidence of association with MS have been described [8–10]. Many of these loci had long been suspected as having a role in MS whilst others were more surprising, but the majority have clear roles in the immune system [8]. Several of the identified genes had been identified as therapeutic targets through earlier work, highlighting the importance of a broad range of molecular and cellular approaches to the analysis of this disease. The way in which the molecular targets of the various investigative approaches and ultimately treatments for MS as reviewed in this single issue are summarised in Table 1 and highlights the growing convergence of thought about MS pathogenesis.

In this issue of IJMS there is a comprehensive review of the environmental factors that have been identified in MS [11]. The molecular mechanisms involved in these environmental factors also shows a clear overlap with the known genetic and pathophysiological mechanisms of MS. Studies are now starting to draw together the known genetic and environment risk factors and plausible biological mechanisms are emerging both at the population level [12] and at the molecular level [13], with evidence that relevant contributions of genetic and environmental factors may be different in different individuals according to geography and potentially other factors. Thus, there is evidence of heterogeneity and complexity in MS at both the population and molecular levels.

Continuing advances in the study of MS at the genetic, molecular and cellular levels has undoubtedly had a significant impact on our understanding of this disease as exemplified by the articles in this special issue of IJMS. This ranges from detailed studies of the pharmacodynamic effects of vitamin D in a single patient [14] through to the detailed analysis of microRNA and their effects on gene expression [15] in what must be one of the most complex biological systems yet discovered. These recent advances in our understanding of biology and disease processes in MS take the notion of complexity to a whole new level. Whilst around 60 genes have been identified as having a role in MS there is evidence to suggest that the total number of genetic loci conferring an increased risk may be 350 to 1500 [12,16]. Whilst many of these may be specific genes, it also seems likely that some may relate to epigenetic effects through transcription factors and microRNA. Getting a firm handle on these effects is going to take considerable effort with carefully defined case material and continued collaborations utilising large datasets. However, as demonstrated by Patel et al detailed studies in both animal models and cell-based studies can shed considerable light upon the biological pathways involved in MS [17]. Indeed, it is notable that many of the examples listed in Table 1 were originally identified as being important in MS and utilized in effective therapies long before the genetic confirmation of their association finally emerged (e.g., VCAM1 and IFNγ). It is also notable that many of the currently non-significantly associated loci included in Table 1 appear very high up on the list of next most associated “hits” from the WTCCC2 experiment [10]. It therefore seems likely that many other molecules will be added to this list in due course as yet larger experiments are completed (e.g., NFκB).

The result of this huge effort in MS research has been the emergence of a variety of effective therapies over the past 25 years [18]. There is little doubt that these treatments have had a significant impact upon the long term outlook for people with MS, despite recent concerns about the long term efficacy of the first disease modifying therapies [19]. All of these therapies have been developed as a result of the study of the immunopathology of MS at the molecular level with several of their therapeutic target molecules being subsequently confirmed as playing a role in susceptibility through the recent large scale genome screens (summarised in Table 1). Whilst the effect size for these genes in their association with MS is generally small (except HLA) with odds ratios typically in the range of 1.1–1.2, this does not mean that they are not mechanistically very important as evidenced by the way in which many of these loci have already been targeted by effective therapies. Furthermore the growing convergence of information relating to common immunological pathways will hopefully also prove fruitful. The identification of many other genes will only accelerate the testing of novel potential therapeutics. Potential drugs targeting many of the genes identified to date have already been identified with preliminary clinical trials being undertaken.

Stem cell therapy offers huge potential to not only alter the course of the inflammatory phase of multiple sclerosis, but to also regenerate damaged central nervous tissue and reverse accumulated disability. As reviewed in this issue of IJMS [20], this potential therapeutic area is making huge leaps forward and mesenchymal stem cell therapy certainly offers a potentially low risk and highly beneficial treatment option [21]. Continued efforts to understand whether it is the immunomodulatory effects or the potential regenerative effects of this treatment modality that accounts for its efficacy are required and this will undoubtedly require a molecular as well as cellular approach. Oligodendrocyte biology [17] and mechanisms of myelin repair [22] are likely to become increasingly important as we not only attempt to prevent the inflammatory insult in MS, but also promote repair of the MS plaque. The prospects for even better treatment for this potentially debilitating disease in the very near future are looking brighter than ever. It is truly an exciting time to be involved in MS research.

## Figures and Tables

**Table 1:** Convergence of molecular targets from investigative and treatment modalities in MS.

Gene	Chr	Environment	Pathophysiology	MicroRNA	Treatment
HLA-DR	6	Vit D deficiency	Antigen presentation		
CD40	20		Antigen presentation		
IL2RA	10		Lymphocyte activation		Daclizumab
CCL3	11		Lymphocyte egress		Fingolimod *
CCL3	17		Lymphocyte egress		Fingolimod *
VCAM1	1		Lymphocyte migration		Natalizumab
STAT1	2		Lymphocyte activation		
IL12A	3		Lymphocyte activation		Glatiramer acetate
IL12B	5		Lymphocyte activation		Glatiramer acetate
IFNγR	6		Lymphocyte activation		β-IFN
TNFα	6		Lymphocyte activation		
STAT3	17		Lymphocyte activation		
IL12RB1	19		Lymphocyte activation		Glatiramer acetate
Nrf2	2		Oligodendrocyte injury		BG12
IL17	6		Reduced acetylcholine	miR-326	
IL10	1	EBV	T cell proliferation		Teriflunomide *
CD52	1		T cell proliferation		Alemtuzumab
NFKB1	4		T cell proliferation	miR-223	Teriflunomide *
IL6	7	Smoking	T cell proliferation		Teriflunomide *
CYP27B1	12	Vit D deficiency	Vitamin D metabolism		Vitamin D
CYP24A1	20	Vit D deficiency	Vitamin D metabolism		Vitamin D

Evidence for association †	
	= *p* < 5 × 10^−8^
	= *p* < 10^−3^
	= ns

* mechanism of action not specific to this molecule but is similar;

† determined by genomewide association screens [8–10]; Vit D = Vitamin D; Chr = chromosome.
